# An engineered antimicrobial peptide Z-FV7 demonstrates bactericidal efficacy against multidrug-resistant *Escherichia coli* in a murine model of endometritis

**DOI:** 10.1186/s13567-025-01593-x

**Published:** 2025-07-25

**Authors:** Jieru Su, Hao Yang, Siyu Wang, Xue Wang, Wei Li, Wenwen Meng, Jiajun Sun, Yaohong Zhu

**Affiliations:** 1 College of Veterinary Medicine, China Agricultural University, Beijing, 100193 China; 2 Sanya Institute of China Agricultural University, Sanya, 572025 China; 3 College of Veterinary Medicine, Inner Mongolia Agricultural University, Hohhot, 010018 China

**Keywords:** Antimicrobial peptides, multidrug-resistant bacteria, *Escherichia coli*, NF-κB signalling pathway, endometritis

## Abstract

Endometritis is a common reproductive disorder in dairy cows, with antibiotics being the primary treatment option. However, the overuse of antibiotics has contributed to the growing problem of antimicrobial resistance. Antimicrobial peptides (AMPs) have been widely studied for their ability to kill bacteria and modulate immune responses. Previous research has focused on modifying natural AMPs extracted from *Zophabas atratus *haemolymph; however, these peptides have displayed limited effectiveness against bacteria. To overcome this limitation, researchers engineered a modified AMP, Z-FV7, by incorporating the sequence of the human-derived AMP LL-37. The resulting peptide demonstrated a favourable safety profile, with a minimum inhibitory concentration (MIC) reduced to 32 μg/mL and improved antibacterial activity against pathogens such as *Staphylococcus aureus* and *Pseudomonas aeruginosa*. Z-FV7 was tested in a bovine uterine epithelial cell model infected with *Escherichia coli* and in a murine model of endometritis. The findings showed that Z-FV7 alleviated clinical symptoms, inhibited the activation of the NF-κB signalling pathway induced by drug-resistant *E. coli*, reduced the expression of pro-inflammatory cytokines (TNF-α, IL-1β, IL-6), and promoted the expression of tight junction proteins (Claudin-1 and ZO-1). These results indicated that Z-FV7 can help reduce uterine inflammation and provide therapeutic outcomes similar to those of antibiotics during *E. coli* infection. Overall, Z-FV7 holds promise as a potential alternative to antibiotics for treating endometriosis in the future.

## Introduction

Antibiotics are the most widely used antimicrobial drugs; however, their effectiveness is declining due to the growing problem of bacterial resistance [1]. As a result, researchers are exploring alternatives to antibiotics, such as antimicrobial peptides.

Antimicrobial peptides are biologically active compounds that can effectively kill bacteria, viruses and other microorganisms. Studies have shown that insects and plants produce these peptides to destroy and protect themselves from pathogenic microorganisms [2].

In addition to their bactericidal properties, antimicrobial peptides can regulate the immune response, modulate cytokine secretion, reduce inflammation, promote tissue and cell recovery, and help establish a balanced microbial community. They also have the potential to reduce the development of microbial resistance due to their unique mechanisms of action, making them viable alternatives to antibiotics [3–5].

Antimicrobial peptides can inhibit bacterial cell wall synthesis and damage cell membranes through various models, such as the barrel-stave model and ring-pore model (transmembrane pore models), as well as the carpet model and detergent-like model (non-transmembrane pore models). This leads to bacterial death, inhibition of biofilm formation, and disruption of nucleic acid synthesis, ultimately reducing the activity of related enzymes [6, 7].

However, natural antimicrobial peptides still have significant limitations in their application. These challenges include low yield, a complicated extraction process, high cytotoxicity, and limited effectiveness against Gram-negative bacteria, which pose the greatest threat in terms of infection [5]. According to the US Centers for Disease Control and Prevention (CDC), secondary infections caused by Gram-negative bacteria result in $45 billion in healthcare costs annually [8].

To address these shortcomings, it is essential to improve natural antimicrobial peptides. This can be achieved by modifying the peptides through various means, such as replacing specific amino acids, adjusting the peptide chain length, altering the peptide’s structure, and implementing chemical modification [9].

These modifications can enhance the effectiveness of antimicrobial peptides, reduce cytotoxicity, improve their anti-enzymatic effects, and increase their efficacy against Gram-negative bacteria. Additionally, they can mitigate the harmful effects of endotoxins (LPS) produced by the peptides on the host [10, 11].

Bovine endometritis is a disease caused by microorganisms that invade the uterus through the open cervix of cows during or after pregnancy. This condition often leads to reproductive disorders and decreased milk production, resulting in significant economic losses [12, 13]. In a study conducted on diseased dairy cattle samples in Ethiopia, *Escherichia coli* was found in nearly 50% of the samples, often in combination with other bacteria. Notably, *E. coli* exhibited multidrug resistance (MDR), showing resistance to antibiotics such as gentamicin, amoxicillin, and ceftiofur [14–16]. The endotoxin LPS produced by *E. coli* triggers the secretion of inflammatory cytokines, affects the function of the hypothalamus and pituitary gland, and disrupts the reproductive cycle [17].

Based on previous studies, we found that *Zophabas atratus * haemolymph is highly susceptible to LPS. *Z. atratus * haemolymph was found to be effective against both Gram-positive and Gram-negative bacteria. However, due to the limitations of natural antimicrobial peptides, we screened defensin antimicrobial peptides for modification. The results of modifying the antimicrobial peptide, Z-d14CFR, showed that its ꞵ-folded special structure significantly enhanced its antimicrobial activity.

Building on this, our laboratory continued to modify the antimicrobial peptide while maintaining the role of the ꞵ-structure in capturing LPS. We also incorporated the sequence of the human antimicrobial peptide LL-37 (FKRIVQRIKDFLR) to improve the peptide’s antimicrobial efficacy and its ability to capture bacterial components, ultimately reducing subsequent damage.

In this experiment, we utilised a newly modified antimicrobial peptide to investigate its therapeutic mechanism for treating clinical *E. coli* endometritis in dairy cows. Our goals were to explore the mechanism of action of the peptide, evaluate its effectiveness as a treatment, and establish a foundation for utilising this antimicrobial peptide in the management of endometriosis in dairy cows.

## Materials and methods

### Bacterial strains and cell lines

Bovine endometrial epithelial cells (BEECs) were used in this experiment, which were preserved at the College of Veterinary Medicine, China Agricultural University (Beijing, China). The cells were cultured in DMEM/F12 (HyClone Inc., USA), supplemented with 10% fetal bovine serum (Thermo Fisher Scientific, USA) and 100 U/mL of penicillin and streptomycin (Invitrogen, Carlsbad, CA, USA). The culture was maintained at 37 °C in a 5% CO_2_ incubator.

Clinical *E. coli* strains were isolated from the uterine contents of cows at Yinchuan Beef Farm, Ningxia, China. A clinically resistant *E. coli* CAU202308 strain was selected for this study. This strain was chosen as the model, as it has shown resistance to commonly used antibiotics, including ciprofloxacin, vancomycin, and cefquinome. Thus, this model allows for the evaluation of the peptide as a potential alternative to antibiotics in bactericidal treatment.

### Peptide design and synthesis

The amino acid sequence of Z-FV7 is H_2_N-Arg-Gly-Cys-Arg-Cys-Asn-Ser-Lys-Ser-Phe-Cys-Val-Cys-Arg-Gly-Gly-Gly-Gly-Ser-Phe-Lys-Arg-Ile-Val-Gln-Arg-Ile-Lys- Asp-Phe-Leu-Lys-Asp-Phe-Leu-Arg-NH_2_, with the molecular formula C_153_H_258_N_56_O_39_S_4_. It was synthesised by Sangyo Bioengineering Chemical Synthesis Co Ltd (Shanghai, China).

### Circular dichroism spectroscopy (CD)

The CD spectra of the peptides in pure water were measured using a JASCO J-1500 circular dichroism spectrometer from Japan, with the molar ellipticity recorded in the range of 180–260 nm.

### Antimicrobial activity

Z-FV7 was diluted to create a concentration gradient ranging from 0.5 to 512 μg/mL, while Z-d14CFR was diluted to achieve a concentration gradient of 100 to 2000 μg/mL using the microdilution method. A volume of 100 μL of each drug was added to each well of the 96-well plate. The bacteria were incubated until the suspension reached OD_600_ = 0.6. The suspension was then diluted to a concentration of 10^8^ CFU/mL using 10 mM PBS. Following this, 100 μL of the bacterial solution was added to each well and incubated for 24 h at 37 °C.

Peptides were determined using the following method. The wells were incubated at 37 °C for 24 h to determine the lowest concentration of peptide and antibiotic that completely inhibits bacterial growth. This formed the MIC (Minimum Inhibitory Concentration).

### Haemolysis test

After centrifuging 5 mL of sheep blood at 8000 rpm for 10 min at 4 °C, the erythrocyte suspension was washed three times with 10 mL of PBS (10 mM). The peptide was diluted in a gradient with PBS in each tube. In separate tubes, equal amounts of PBS and 0.2% Triton X-100 were added to serve as negative and positive controls, respectively. These samples were then incubated at 37 °C for 1 h. Following incubation, the OD value of the supernatant was measured at 570 nm.

### Cytotoxicity

Stable bovine endometrial epithelial cells (BEECs) were selected, and the cell suspension was added to 96-well plates, at about 10^4^ cells per well. The plates were then incubated at 37 ℃ for 12 h. The medium was washed with PBS (10 mM), and then 100 µL of gradient PBS diluted Z-FV7 (0.25–32 × MIC) was added to each well in turn. 100 µL of PBS was added to the control wells, and the cells were incubated at 37 ℃ for 12 h. After incubation, 10 µL CCK8 solution was added to each well. Incubation continued at 37 °C for 4 h, and the OD value was determined at 450 nm. Cell viability (%) = (OD_450_Peptide- OD_450_Blank)/(OD_450_PBS- OD_450_Blank) × 100%.

### Cell viability test

Stable dairy endometrial epithelial cells (BEECs) were selected, and the cell suspension was added to 96-well plates, about 10^4^ cells per well. They were then incubated at 37 ℃ for 12 h. The medium was next washed with PBS (10 mM), and the gradient PBS diluted Z-FV7 (1–16 × MIC) was added, 100 µL per well in sequence. 100 µL PBS was then added to the control wells and incubated at 37 °C for 12 h. The Calcein/PI cell activity and cytotoxicity assay kit was used for the assay, and the stained cells were observed and photographed using a fluorescence microscope.

### Bacterial growth curve

The bacteria were cultured until the suspension reached OD_600_ = 0.6 and then washed with PBS (10 mM) once. They were then diluted with LB medium until the OD reached 0.12 (about 10^8^ CFU/mL), and then incubated at a constant temperature of 37 ℃ for 24 h. After incubation, the OD values of each time point were collected, and the growth curve was plotted.

### Time-killing assay

In a 96-well plate, the peptide (0.5–64 × MIC) was diluted with a PBS gradient, and 100 µL of each dilution was added to each well. The bacteria were incubated until the suspension reached OD_600_ = 0.6. The wells were then washed three times with PBS and diluted with PBS until the OD reached 0.24. Following this, 100 µL of the bacterial suspension was added to each well (the concentration of the bacteria in the wells was about 10^8^ CFU/mL). The plate was incubated for 24 h at a constant temperature of 37 °C in a microplate reader. After a 24-h incubation, the OD values were recorded at each time point, and the bactericidal curve was plotted. An aliquot of 20 µL of the bacterial solution was spread on LB agar medium and incubated at 37 °C for 24 h. The minimum drug concentration that can reduce the number of colonies below 10 CFU is the MBC.

### Bacterial viability staining test

A gradient dilution of the peptide Z-FV7 (0.5–8 × MIC) was prepared in 10 mM PBS, and 100 µL was added to each well of a 96-well plate. Bacterial cultures were incubated at 37 °C until the suspension reached an OD_600_ of 0.6, then washed once with PBS and diluted in LB medium to an OD_600_ of 0.12 (approximately 10^8^ CFU/mL). Subsequently, 100 µL of the bacterial suspension was added to each well and incubated for 3 h. After incubation, a 1:2 mixture of SYTO-9 and PI was added to each well and incubated for an additional 30 min. The samples were then re-suspended in ddH_2_O. Then, 10 µL of the suspension was placed onto a microscope slide, air-dried, and fixed with neutral resin. Finally, the bacterial cells were observed and imaged using a Nikon A1 confocal laser scanning microscope.

### Bacterial biofilm assay

The peptide was serially diluted in PBS (0.5–64 × MIC), and 100 µL of each concentration was added to wells of a 96-well plate. Polymyxin B (4 µg/mL) was used as a positive control, and untreated *E. coli* cultures served as a negative control. Bacteria were grown to an OD_600_ of 0.6, diluted to 10^5^ CFU/mL in LB medium, and 100 µL of the suspension was added to each well. After a 48-h incubation, wells were rinsed once with PBS and fixed with 4% paraformaldehyde. Fixed samples were stained with 0.1% crystal violet for 30 min, followed by rinsing with distilled water. Staining was decolorized using 95% ethanol, and absorbance was measured at 570 nm. Finally, the biofilm destruction rate was calculated as follows: Destruction rate % = (OD control group—OD experimental group)/OD control group × 100%.

### Cell infection model

Bovine endometrial epithelial cells (BEECs) in a stable state after passaging were grouped together (*n* = 5). The cells were divided into several groups: the attack group, the treatment group, and the polymyxin B treatment group. Each of these groups received a 20 µL suspension of *E. coli* CAU202308 (MOI = 10) mixed with 2 mL of DMEM/F12. The control group and the antimicrobial peptide alone treatment group were given only 2 mL DMEM/F12.

After incubating at 37 ℃ for 1 h, 20 µL of Z-FV7 (64 µg/mL) was added to both the treatment group and the antimicrobial peptide alone treatment group. Additionally, 20 µL of polymyxin B (4 µg/mL) was added to the polymyxin B treatment group. The mixtures were thoroughly mixed and then incubated at 37 °C for an additional 2 h.

After a three-hour incubation, samples were collected for assays of inflammatory factors, immunofluorescence, and protein immunoblotting.

### Mouse infection model

All animals used in this study were bred and managed in strict compliance with Chinese legislative frameworks and regulations (Protocol GK-FCZ2001545), the EU Directive 2010/63/EU on animal experimentation, and the guidelines set by China Agricultural University concerning the ethical treatment of animals for scientific purposes (2010-SYXK-0037).

Animal welfare during the mouse experiments was strictly adhered to, following the Implementing Rules for the Management of Laboratory Animals, which were approved by the Ethical Review Committee for Laboratory Animal Welfare and Animal Experiments of China Agricultural University (AW05903202-2-1). A total of 45 six-week-old female mice were purchased from Beijing Viton Lihua Laboratory Animal Technology Co. The mice were randomly divided into five groups (*n* = 9) and anaesthetised using Sutent^®^.

After anaesthetising the mice, each one was lifted by its tail and received an injection into its uterus using a round-tipped syringe. The mice in the attack group, treatment group and polymyxin B treatment group received an injection of *E. coli* CAU202308 suspension 25 µL/pupil (1 × 10^8^ CFU). The control group and the antimicrobial peptide-alone group were injected with saline, 25 µL/pupil.

Twelve hours later, the treatment group and the peptide-alone group were given an additional injection of 25 µL/pupil of Z-FV7 (32 µg/mL). The polymyxin B treatment group received 25 µL/pupil (2 µg/mL), while the remaining groups were injected with 25 µL/pupil of saline and *E. coli* CAU202308 suspension of 25 µL/pupil.

After another twelve hours, uterine tissues from the mice were collected for weight determination, bacterial counts, histopathological examination, detection of inflammatory factors, immunofluorescence, and protein immunoblotting tests.

### Uterine bacterial burden

0.1 g of uterine tissue was placed in a pre-cooled EP tube, and 900 µL of 10 mM PBS was added. The samples were homogenized using a microtissue grinder (45 MHz, 90 s), then centrifuged at 12 000 rpm for 15 min at 4 °C. The supernatant was serially diluted from 10^–1^ to 10^**–**8^, and 20 µL of each dilution was plated onto LB agar. Plates were incubated overnight at 37 °C, and bacterial colonies were counted.

### Histopathological examination

Uterine tissues were fixed in 4% paraformaldehyde for 72 h, then dehydrated and embedded in paraffin wax. The tissues were sliced into 3 µm sections, stained, and sealed with neutral resin as described. Finally, the samples were observed under a light microscope [18].

### Inflammatory factor assay

Total RNA was extracted from uterine tissues and BEECs cells using RNAiso Plus (TaKaRa, Japan). For reverse transcription of RNA, we employed the TransScript All-in-One First-Strand cDNA Synthesis SuperMix for PCR (TransGen Biotech, China). Real-time quantitative PCR was conducted using the TransScript Green mRNA Two-Step RT-qPCR SuperMix (TransGen Biotech, China). The reaction conditions included pre-denaturation at 95 °C for 30 s, denaturation at 95 °C for 10 s, and annealing at 58 °C for 30 s, repeated for 45 cycles. The primer details are listed in Table 1. GAPDH was used as the internal reference gene, and the gene expression levels were analysed using the 2^−ΔΔCT^ method.

**Table 1:** **Primers sequences of qPCR**

Gene	Forward primer (5′-3′)	Reverse primer (5′-3′)
GAPDH (cattle)	GTCTTCACTACCATGGAGAAGG	TCATGGATGACCTTGGCCAG
IL-1ꞵ (cattle)	CCTCGTTCCATGGGAGATG	AGGCACTGTTCCTCAGCTTC
IL-10 (cattle)	GTTGCCAAGCCTTATCGGAA	TCAGGCCCGTGGTTCTCA
IL-6 (cattle)	TTCCATCCAGTTGCCTTCTT	CAGAATTGCCATTGCACAAC
TNF-α (cattle)	TCCAGAAGTTGCTTGTGCCT	CAGAGGGCTGTTGATGGAGG
GAPDH (mouse)	GCTCTTTTCCAGCCTTCCTT	GATGCAACGTCACACTT
IL-1ꞵ (mouse)	TGCCTTTGACAGTGATG	AAGGTCCACGGGAAAGACAC
IL-10 (mouse)	GTTGCCAAGCCTTATCGGAA	GAGGGTCTTCAGCTTCTCACC
IL-6 (mouse)	TTCCATCCAGTTGCCTTCTT	CAGAATTGCCATTGCACAAC
TNF-α (mouse)	ACGGCATGATCTCAAAGAC	AGATAGCAAATCGGCTGACG

### Immunofluorescence

BEEC cells were fixed with 4% paraformaldehyde for 15 min and subsequently permeabilised with 1% Triton X-100. Following permeabilisation, the cells were blocked with 2% BSA for 1 h. They were then incubated overnight at 4 °C with TLR4 (1:100 dilution, Proteintech), P-NF-κB p65 (1:100 dilution, ABclonal), ZO-1 (1:100 dilution, Proteintech), and Claudin-1 (1:100 dilution, Proteintech). Cells were incubated with secondary antibodies for 1 h at room temperature, protected from light. Nuclei were stained with DAPI (C0065, Beijing Solepol Technology Co., Ltd.). BEEC cells were observed and photographed using a Nikon A1 confocal laser scanning microscope.

Tissue sections were first dewaxed and rehydrated, followed by antigen retrieval using a citrate buffer solution. After three washes with 10 mM PBS, the sections were blocked with 10% goat serum for 1 h. Primary antibodies, including TLR4 (1:100 dilution, Proteintech), P-NF-κB p65 (1:100 dilution, ABclonal), ZO-1 (1:100 dilution, Proteintech), and Claudin-1 (1:100 dilution, Proteintech), were diluted in PBS containing 5% goat serum and 0.3% Triton X-100. The sections were then incubated overnight at 4 °C with the primary antibodies.

Afterwards, secondary antibodies were applied for 1 h at room temperature in the dark. Nuclei were stained with DAPI (C0065, Beijing Solepol Technology Co., Ltd.). Imaging of bovine endometrial epithelial cells (BEECs) and the tissue sections was performed using a Nikon A1 confocal laser scanning microscope. The fluorescence intensity of the tissue sections was quantified using Image J.

### Western blotting

Cells were lysed with 1 mL of RIPA lysate supplemented with 10 µL of phenylmethylsulfonyl fluoride (Solarbio, Beijing, China). The lysate was then centrifuged at 12 000 x *g* for 10 min at 4 °C. The supernatant was collected to determine the protein concentration. The measured proteins were subjected to electrophoresis and subsequently transferred to a PVDF membrane. After blocking the membrane, it was incubated overnight with the primary antibody listed in Table 2. Following this incubation, the membrane was again blocked and then incubated overnight with the primary antibody. It was then incubated with a secondary antibody (as shown in Table 2) for 45 min. The membrane was then dipped into an ECL ultrasensitive luminescent solution and developed using the Tanon 6200 Chemiluminescence Imaging Workstation (Tanon, Shanghai, China). Finally, the membrane was photographed, and the optical density values were determined using ImageJ.

**Table 2:** **Antibodies related to protein immunoblotting analysis**

Name	Dilution rate	Manufacturers
TLR4	1:1000	Proteintech
MyD88	1:1000	CST
IκBα	1:1000	Proteintech
Phospho- IκBα	1:1000	Abcam
NF-κB p65	1:1000	Proteintech
Phospho-NF-κB p65	1:1000	ABclonal
Claudin-1	1:1000	Proteintech
ZO-1	1:1000	Proteintech

For the mouse uterus, 1 g of uterine tissue was added to 1 ml of RIPA lysate. The mixture was homogenised for 90 s using a homogeniser set to 45 MHz. After homogenisation, the lysate was centrifuged at 12 000 x *g* for 15 min at 4 °C. Following centrifugation, the subsequent steps were carried out in the same manner as for the cells.

### Statistical analysis

GraphPad Prism 9.2.0, SPSS 26.0, and Origin 9.8.0 were utilised for statistical analysis. All data are presented as mean ± standard deviation (SD). Significance analysis was conducted using one-way ANOVA with Tukey's multiple comparison test, *P < 0.05, **P < 0.01, ***P < 0.001.

## Results

### Peptide design and structure analysis

**Figure 1:**
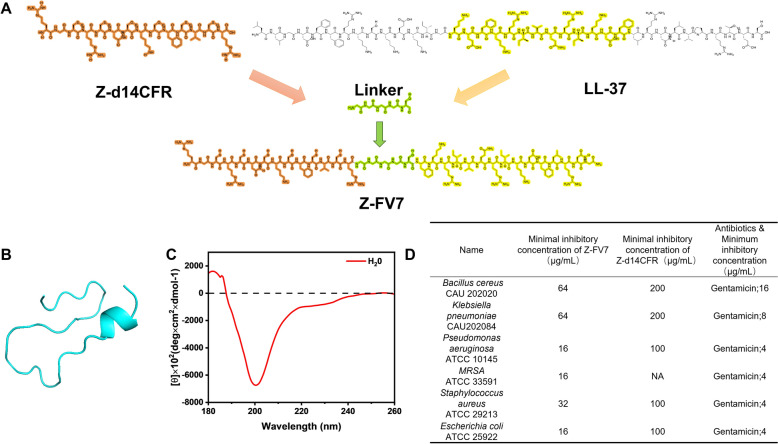
**Peptide design and structure analysis.** **A** Schematic structure of Z-FV7 synthesis. **B** Tertiary structure prediction of the antimicrobial peptide Z-FV7. **C** Circular dichroism (CD) spectra of Z-FV7 in pure water. **D** MIC values of the antimicrobial peptide Z-FV7 and Z-d14CFR against different bacteria. Gentamicin was used as a positive control. NA indicates that the concentration of the drug, less than 1 mg/mL, has no antimicrobial effect. All experiments were repeated three times.

In this experiment, we modified an antimicrobial peptide to enhance its antimicrobial activity compared to the original antimicrobial peptide. We achieved this by adding the antimicrobial peptide sequence LL-37 to the tail end of the protein. LL-37 is an endogenous antimicrobial peptide found in rats, mice, and humans, possessing antimicrobial properties. The original antimicrobial peptide and LL-37 are connected by a flexible linker sequence, GGGGS (Figure 1A), which helps resist protease hydrolysis and protects the functional activity of both ends of the protein.

Next, we predicted the three-dimensional structure of the peptide using the I-TASSER program. The expected structure indicated that Z-FV7 formed a ꞵ-folded structure, while LL-37 partially retained its intrinsic α-helical structure (Figure 1B). This structure is partially due to LL-37 itself, which also exhibits antibacterial activity [19].

To obtain the precise structure of this peptide, we measured the CD spectra of Z-FV7 in pure water. The results showed a peak at 200 nm (Figure 1C), confirming that Z-FV7 formed an unconventional ꞵ-hairpin structure in pure water. We then determined the MICs of Z-FV7 against *Staphylococcus aureus* and other bacteria. The results demonstrated that compared to the original antimicrobial peptide Z-d14CFR, the modified peptide Z-FV7 exhibited enhanced broad-spectrum antimicrobial activity, with lower MIC values and improved effectiveness in inhibiting bacterial growth (Figure 1D).

### Safety test of Z-FV7

**Figure 2:**
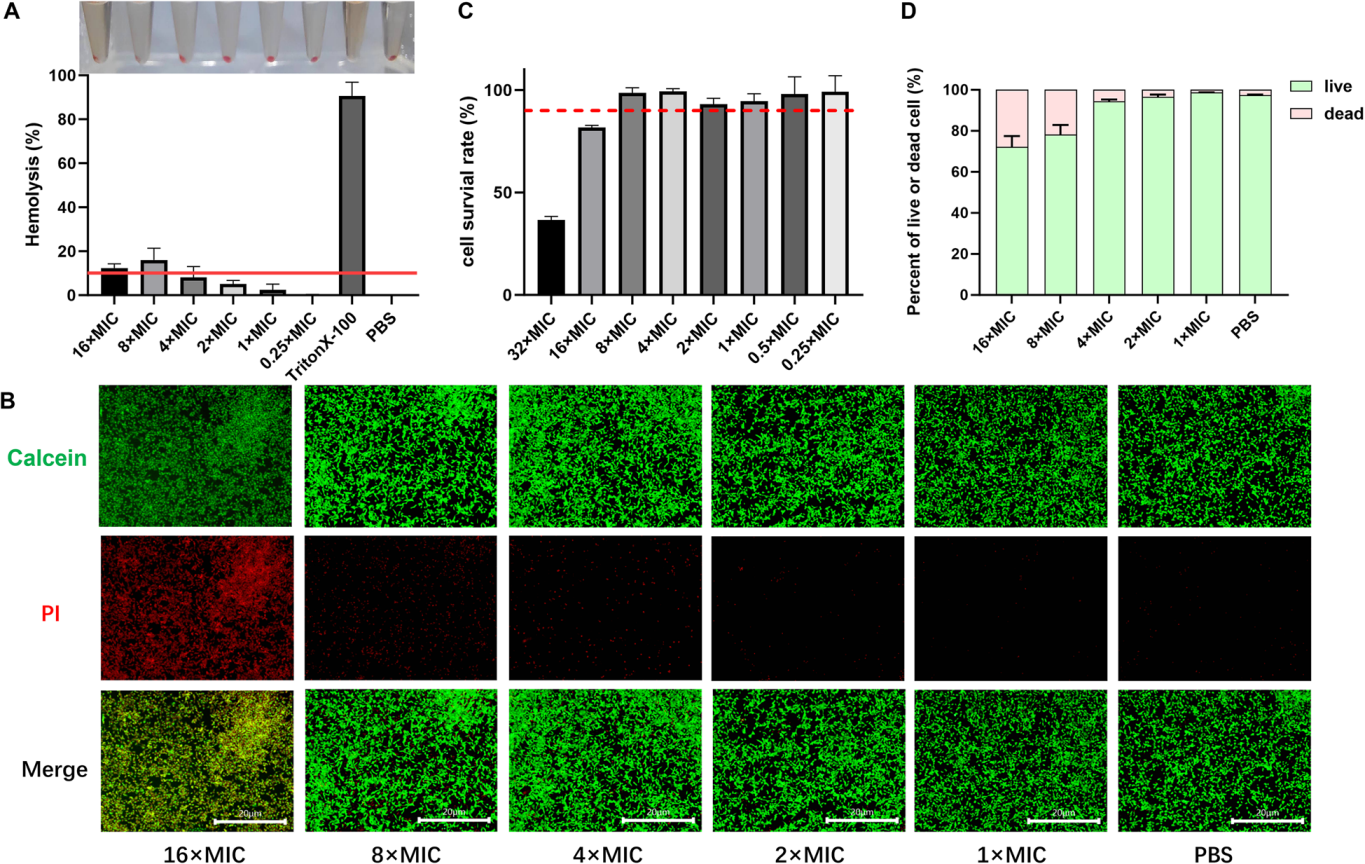
**Safety tests of Z-FV7.** **A** Hemolytic effect of Z-FV7. **B** Fluorescence microscopic Calcein/PI staining of Z-FV7-treated BEEC, red: dead cells, green: live cells. Scale bar: 20 μm. **C** Cytotoxicity of Z-FV7 on BEEC. **D** Damaging effects of Z-FV7 on BEEC. All the results are represented as mean ± SD (n = 3).

In this experiment, we investigated the haemolytic effect of different MIC concentration levels of Z-FV7 on sheep blood erythrocytes. The results showed that the haemolysis rate of erythrocytes remained below 10% at concentrations ranging from 0.25 to 4 × MIC, indicating that Z-FV7 does not exhibit haemolytic activity (Figure 2A).

Additionally, Calcein/PI staining revealed a gradual decrease in the survival rate of bovine endometrial epithelial cells (BEEC) as the concentration of Z-FV7 increased (Figure 2B). Both the CCK-8 assay and Calcein/PI staining indicated that the survival rate of BEEC remained above 90% when exposed to Z-FV7 concentrations between 0.5 and 4 × MIC (Figures 2C and D).

These findings suggest that Z-FV7 shows no significant cytotoxicity toward BEECs, highlighting its potential for clinical use. Moreover, at therapeutic concentrations commonly used in clinical settings, Z-FV7 exhibited minimal cytotoxicity, underscoring its favourable safety profile. Therefore, this antimicrobial peptide is considered very safe for clinical application, with no apparent damaging effects on cells.

### Antibacterial activity test of Z-FV7

**Figure 3:**
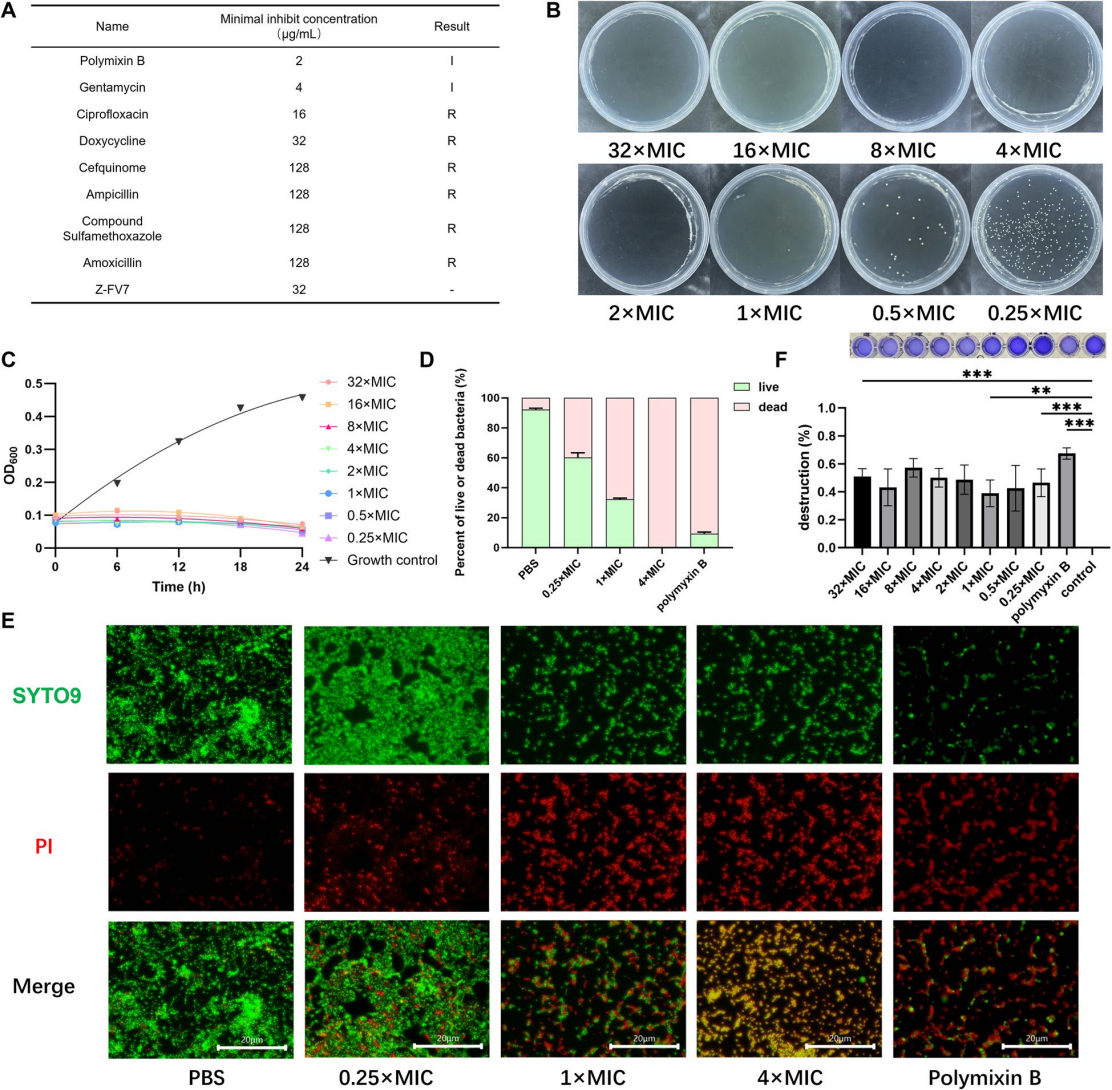
**Antimicrobial activity assay of Z-FV7.** **A** MIC of antibiotics against E. coli CAU202308. “R” means bacteria resistant to the drug, “I” means bacteria moderately sensitive to the drug (according to Clinical and Laboratory Standards Institute M100). **B** MBC of Z-FV7 measured after 24 h. **C** Time-killing curves of 0.25 - 32 × MIC Z-FV7 on E. coli CAU202308. **D** Killing effect of Z-FV7 and polymyxin B against E. coli CAU202308. **E** Biofilm formation in Z-FV7-treated E. coli CAU202308 under fluorescence microscope SYTO9/PI staining, red: dead bacteria, green: live bacteria. Scale bar: 20 μm. **F** 0.25 - 32 × MIC Z-FV7, 4 µg/mL polymyxin B, and 10 mM PBS-treated E. coli CAU202308 biofilm formation. All the results are represented as mean ± SD (n = 3). P values are determined by one-way ANOVA with Tukey’s multiple comparisons test, ** P < 0.01, *** P < 0.001.

In this assay, we utilised *E. coli* isolated from cows clinically diagnosed with endometritis. The growth rate of *E. coli* CAU202308 was measured, reaching a plateau at approximately 12 h. The MIC of *E. coli* CAU202308 was determined, revealing that this strain was resistant to ciprofloxacin, cefquinome, ampicillin, compound sulfamethoxazole, and amoxicillin. It showed moderate sensitivity to polymyxin B and gentamicin (Figure 3A), indicating an increasing resistance to conventional antibiotics, rendering some ineffective for treating infections caused by *E. coli*.

The strain was sensitive to Z-FV7, which demonstrated a significant antibacterial effect with a MIC value of 32 µg/mL. To further investigate the efficacy of Z-FV7 against *E. coli* CAU202308, we assessed its effects at various MIC concentrations. The results indicated that bacterial growth was inhibited from 0.25 × MIC to 32 × MIC. Between 1 × MIC and 32 × MIC, Z-FV7 was able to completely kill the bacteria (Figures 3B and C).

In the bacterial death test, the killing rate reached 70% at the MIC values (Figures 3D and E), with total bacterial elimination occurring at 4 × MIC. Antimicrobial peptides act by disrupting bacterial membranes; therefore, we evaluated the disruption rate of the bacterial membrane by Z-FV7. The results indicated that the membrane disruption rate was 50% at a concentration of 2 × MIC (Figure 3F), suggesting that Z-FV7 also acts through this mechanism.

### Anti-infective effect of Z-FV7 on *E. coli*-infected BEEC cell model

**Figure 4:**
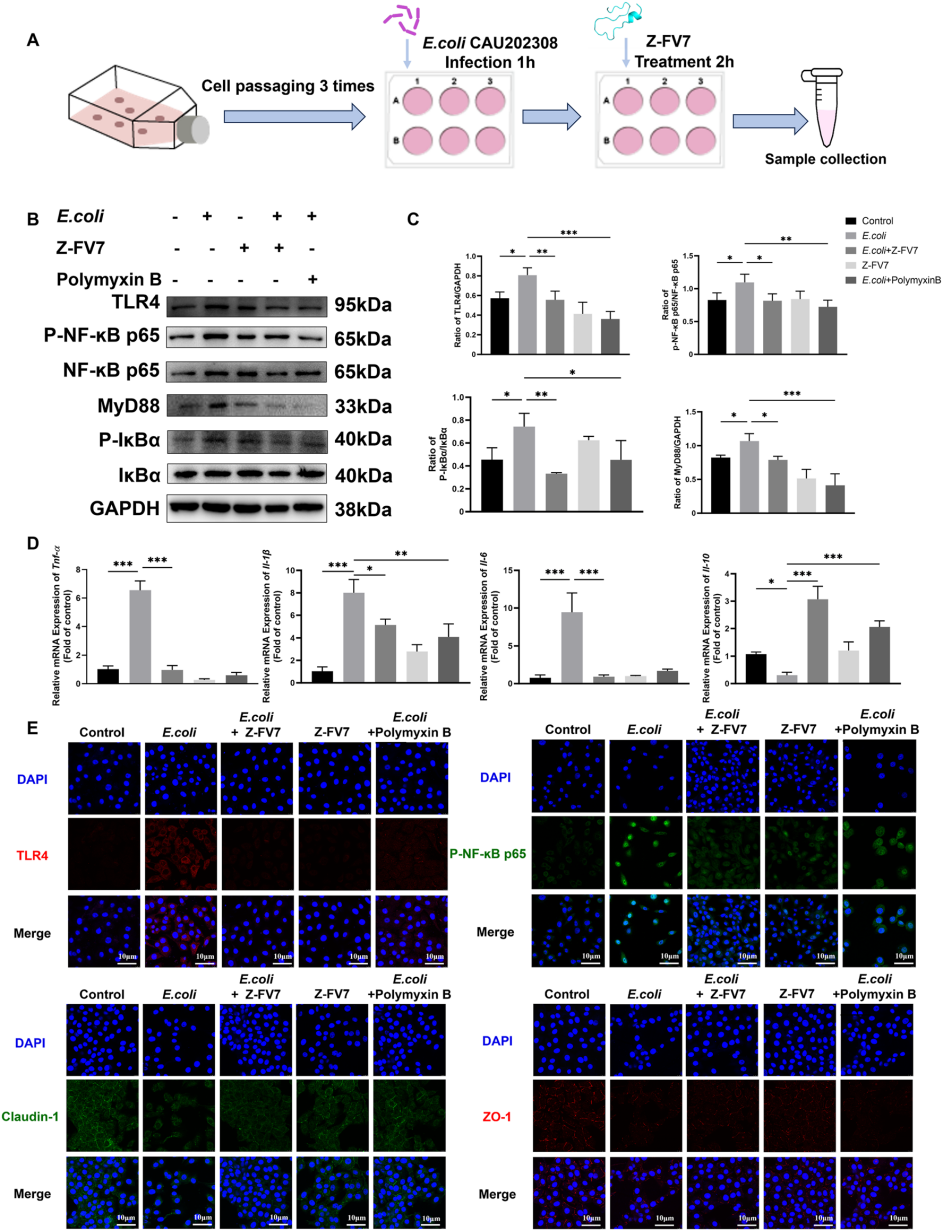
**Anti-infective effect of Z-FV7** **on the BEEC cell model** of ***E. coli*** **infection**. BEEC cells were treated with *E. coli* (MOI = 10) and cells were treated in the presence (+) or absence (-) of Z-FV7 (32 μg/mL) and in the presence (+) or absence (-) of polymyxin B (2 μg/mL). **A** Experimental methods for BEEC cell model construction. **B** Western blot detection of TLR4, MyD88, P-NF-κB-p65, p65, P-IκBα, IκBα in BEEC. **C** Relative protein level of TLR4, MyD88, P-NF-κB-p65, p65, P-IκBα, IκBα. **D** *Tnf-α*, *Il-1ꞵ*, *Il-6*, *Il-10* mRNA level. **E** Immunofluorescence assay to detect the expression of TLR4, P-NF-κB-p65, Claudin-1, and ZO-1 in cells. All the results are represented as mean ± SD (*n* = 3). *P* values are determined by one-way ANOVA with Tukey’s multiple comparisons test, * *P* < 0.05, ** *P* < 0.01, *** *P* < 0.001.

We utilised *E. coli* CAU202308 to challenge the BEEC endometrial epithelial cells of dairy cows under optimal growth conditions. Following 1 h of bacterial exposure, the inflammatory cells were treated with Z-FV7. Cell samples were collected after 3 h of treatment (Fig. 4A), and the results were analysed.

We detected proteins associated with the NF-κB pathway, and western blot results revealed that in the bacterial challenge group, the expression of proteins such as TLR4, MYD88, P-NF-κB-p65, and p-IκBα was significantly upregulated. In contrast, the expression of these proteins decreased in the treatment group, including those treated with polymyxin B. This suggests that the antimicrobial peptide Z-FV7 can inhibit the activation of the classical inflammatory NF-κB signalling pathway (Figures 4B and C) and has effects comparable to traditional antibiotics. Furthermore, there was no notable activation of the inflammatory pathway in the antibiotic group alone, indicating that the antibiotic peptide itself did not induce inflammation.

Next, we examined the expression of intracellular inflammatory factors. Bacterial infection led to a significant increase in the levels of pro-inflammatory factors, such as *TNF-α*, *Il-1ꞵ*, and *IL-6*, while the level of the anti-inflammatory factor *IL-10* decreased. In contrast, treatment with the antimicrobial peptide brought the cytokine levels closer to normal values (Figure 4D). This behaviour can be attributed to the ꞵ-hairpin structure formed by the antimicrobial peptide, which interlocks to create a nano-network that captures the bacteria and the endotoxin LPS produced by the bacteria after they are killed, thus preventing further inflammation.

Cellular immunofluorescence result (Figure 4E) also confirmed that treatment with antimicrobial peptides could inhibit the expression of TLR4 and P-NF-κB-p65. Simultaneously, antimicrobial peptides promoted the expression of tight junction proteins such as Claudin-1 and ZO-1, helping to restore cellular tight junctions and maintain their normal morphology.

### Anti-inflammatory effect of Z-FV7 on *E. coli*-induced endometritis model in mice

**Figure 5:**
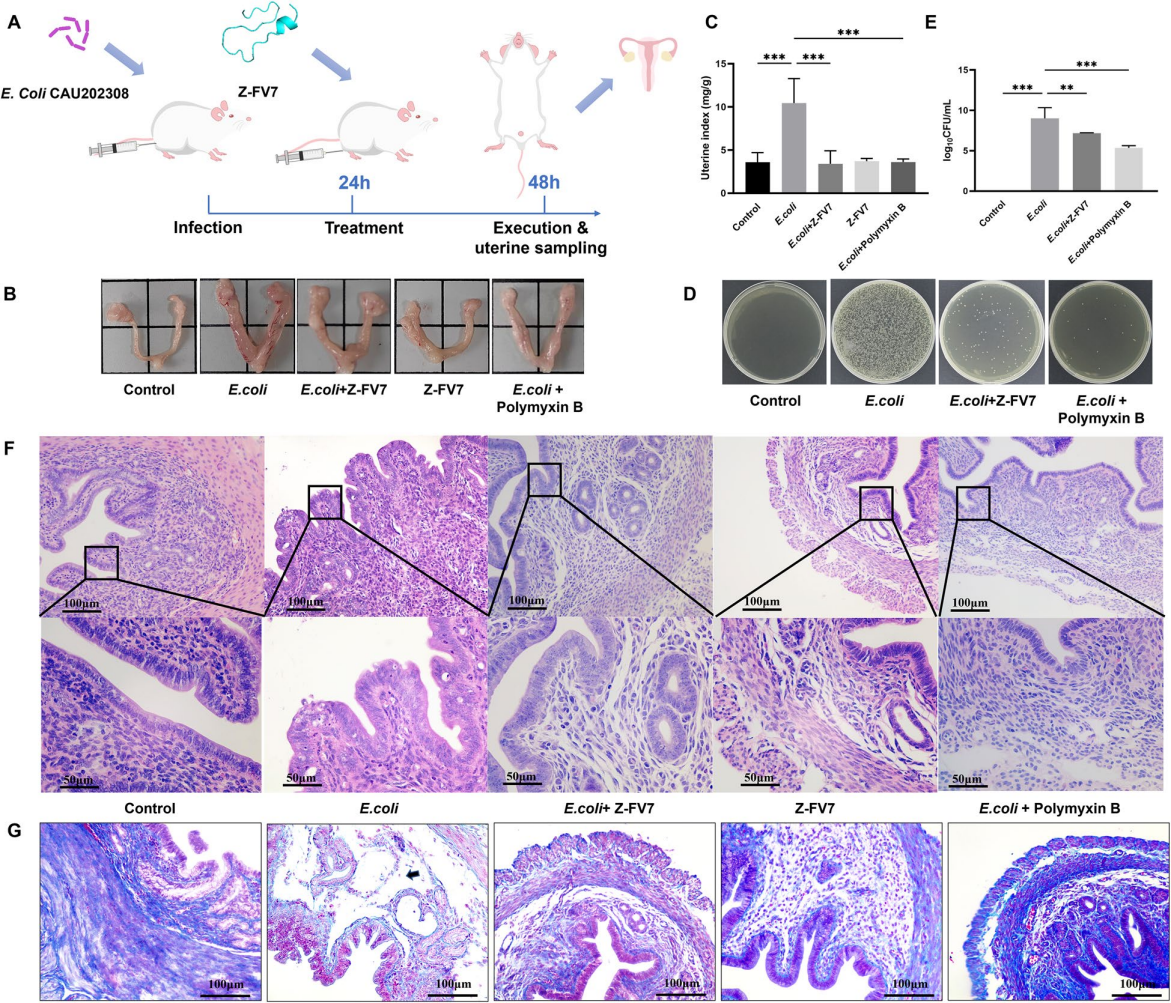
**Anti-inflammatory effect of Z-FV7 on *E. coli*-induced endometritis model in mice.** **A** Experimental method for the construction of a mouse endometritis model. **B** Pictures of mouse uterine tissue. **C** Mouse uterine index. **D** Bacterial culture of mouse uterus tissue. **E** Bacterial load of mouse uterus tissue. **F** H&E staining of uterine histopathology. **G** Masson staining for uterine histopathology. Black arrows indicate reduced collagen fibre density. All the results are represented as mean ± SD (n = 3). ** P < 0.01, *** P < 0.001.

To assess the clinical application potential of Z-FV7, we established an *E. coli*-induced endometritis model in mice. Z-FV7 was administered via perfusion into the uterus to evaluate its therapeutic effects on endometritis (Figure 5A). Upon dissection of the uteri, the endometriosis model group exhibited noticeable congestion and oedema (Figure 5B). Following treatment with Z-FV7, the congestion and oedema in the uterine tissue of the mice were significantly reduced, and correspondingly, the uterine index also decreased (Figure 5C). This indicates that the antimicrobial peptide can alleviate the clinical symptoms of endometritis in these mice.

Furthermore, the antimicrobial peptides reduced the bacterial load in the tissues (Figure 5D). Compared to the infected group, the bacterial load in the group treated with the antimicrobial peptide was reduced to 1.78 × 10^9^ CFU/mL, and the bacterial load in the polymyxin b-treated group was also significantly reduced (Figure 5E). The findings from H&E staining and Masson staining were consistent with these results. In the infected group, we observed vacuolar degeneration in the cells, and some mucosal surface epithelial cells were sloughed off. Additionally, oedema appeared around the glandular ducts, and inflammatory cell infiltration was noted in those ducts. The capillaries were dilated and filled with blood, resulting in scattered bleeding and the presence of red blood cells in the interstitial spaces. The distribution of collagen fibres was relatively sparse. (Figures 5F and G). After treatment with the antimicrobial peptide, these symptoms were significantly alleviated: vacuolisation of cells was reduced, neutrophil infiltration decreased, and collagen fibres had returned to normal.

**Figure 6:**
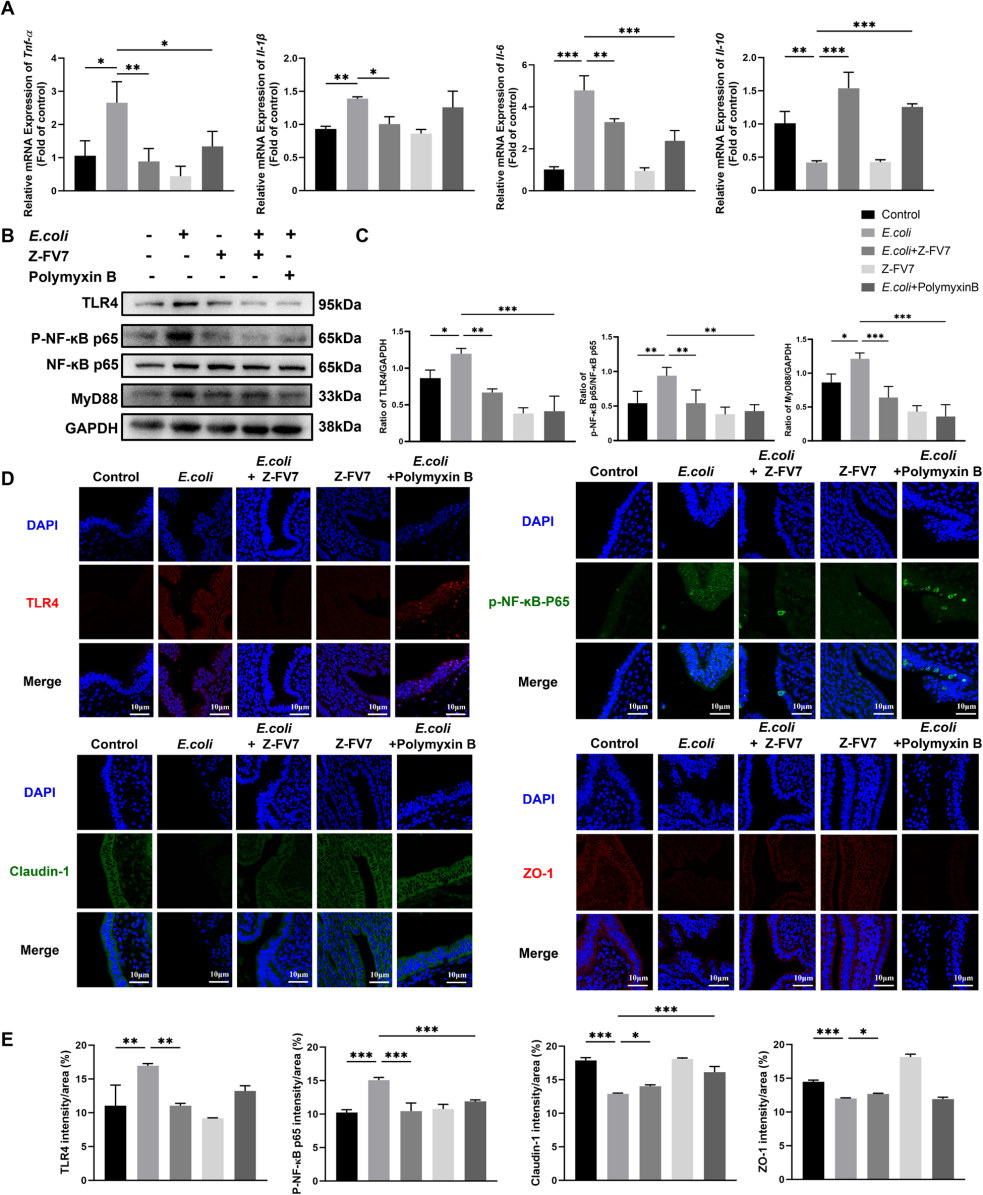
**Inhibitory effect of Z-FV7 on NF-κB signalling pathway in** ***E. coli*-induced endometritis model in mice.** **A** *Tnf-α*, *Il-1ꞵ*, *Il-6*, *Il-10* mRNA level. **B** Western blot detection of TLR4, MYD88, P-NF-κB-p65, p65. **C** Protein levels of TLR4, MYD88, P-NF-κB-p65, and p65 in the *E. coli*-induced endometritis model in mice. **D** Expression of TLR4, P-NF-κB-p65, Claudin-1, and ZO-1 in mouse uterine tissues by immunofluorescence. **E** Immunofluorescence quantification of TLR4, P-NF-κB-p65, Claudin-1 and ZO-1 in mouse uterine tissues. All the results are represented as mean ± SD (*n* = 3). *P* values are determined by one-way ANOVA with Tukey’s multiple comparisons test, *P < 0.05, **P < 0.01, ***P < 0.001.

By further examination of cytokine levels, the results showed that the antimicrobial peptide effectively promoted the production of anti-inflammatory factors while inhibiting the expression of pro-inflammatory factors (Figure 6A). Similarly to the outcomes observed in cellular experiments, the mouse model of endometritis showed that the expression levels of NF-κB pathway-associated proteins, including TLR4, MYD88, and P-NF-κB-p65, were significantly reduced in the treatment group compared to the bacterial challenge group (Figures 6B and C). This suggests a suppression of inflammation. These findings are consistent with the findings from the immunofluorescence analysis (Figures 6D and E). Figure 6D demonstrates that the fluorescence intensity of the tight junction proteins ZO-1 and Claudin-1 was increased in the treatment group compared to the bacterial challenge group, indicating a significant rise in their expression (Figure 6E).

## Discussion

This study systematically investigated the therapeutic efficacy of the antimicrobial peptide Z-FV7 in treating *E. coli*-induced endometritis, with a focus on elucidating its underlying mechanism of action. The original antimicrobial peptide, Z-d14CFR, also showed significant bactericidal activity; however, its MIC was 100 µg/mL, which notably increased treatment costs [20].

To address this limitation, a combination therapy using both antimicrobial peptides was implemented to enhance antimicrobial efficacy, lower the MIC, and thus reduce the overall treatment costs. For the selection of an additional antimicrobial peptide, we chose LL-37, which plays an important immunological role in the human body, with the hope that it would also be effective in other mammals [21].

Currently, LL-37 has demonstrated antibacterial activity against both Gram-negative and Gram-positive bacteria, and it can impact biofilm formation [22, 23]. Additionally, LL-37 has been shown to be effective in inhibiting the growth of dormant *E. coli*, which is responsible for persistent infections. This makes LL-37 particularly effective in the treatment of chronic endometritis caused by *E. coli* [24].

To link the two peptides, we selected the flexible “GS linker” due to its ability to provide enhanced structural flexibility while maintaining a relatively short length, which helps reduce synthesis costs [25]. However, a significant limitation of this linker is its inability to fully separate the two termini of the protein, which may lead to structural alterations caused by interactions between the termini. These interactions can alter the overall peptide structure.

CD spectroscopy results indicated that the peptide still formed a ꞵ-fold structure. This high percentage of ꞵ-fold may explain the lack of a significant α-helix in the results; however, it did not diminish the impact of the added sequence on enhancing antimicrobial activity, which aligns with our experimental findings. Additionally, the MIC value of the peptide was reduced to 32 µg/mL, significantly reducing the amount of the drug required.

Building on this foundation, we further investigated the safety profile of the peptide. Traditional antimicrobial peptides are often linked to high cytotoxicity; however, in this study, we utilised LL-37, an endogenous antimicrobial peptide naturally found in the human body. To optimise its properties, we shortened the length of LL-37 during the modification process, which reduced both production costs and cytotoxicity [26]. LL-37 does not harm cells, significantly lowering the risk of damage to normal cells while enhancing its antimicrobial efficacy.

One mechanism by which antimicrobial peptides kill bacteria involves accumulating negative charges on the bacterial cell membrane and forming holes in the membrane surface [7]. Therefore, the increased peptide enhances the positive charge, allowing it to selectively attract and destroy the negatively charged bacteria on the cell membrane’s surface. The results also indicated that Z-FV7 has a favourable safety profile, with a haemolysis rate of less than 10% and a mammalian cytotoxicity rate of 10% within the applied dose range.

**Figure 7:**
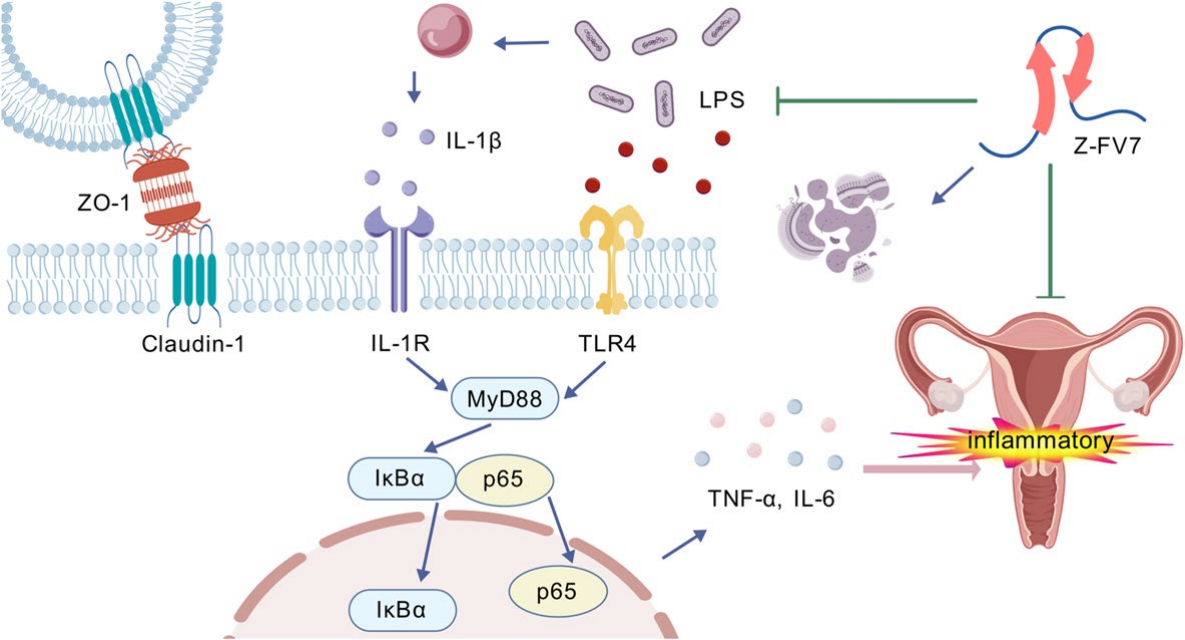
**Mechanism of Z-FV7’s anti-infective action.** Created with BioGDP [36].

*E. coli* is a common pathogen associated with endometrial infections, and the NF-κB signalling pathway is recognised as a key component of the inflammatory response (Figure 7). Numerous studies have demonstrated that lipopolysaccharide (LPS), a primary component of the *E. coli* outer membrane, activates this pathway, resulting in the induction of inflammation. This study evaluated the therapeutic efficacy of the antimicrobial peptide Z-FV7 in bovine endometrial epithelial cells infected with *E. coli* and in a mouse model of endometritis.

In the cellular model, administration of Z-FV7 alone did not induce inflammation. However, Z-FV7 demonstrated the capability to bind to LPS, which is derived from *E. coli*. The binding of LPS to TLR4 resulted in increased expression of TLR4, subsequently activating downstream pathways such as the NF-κB signalling cascade [27–29]. This activation enhanced the expression of inflammation-related proteins, leading to gene transcription within the nucleus and the subsequent release of inflammatory factors. These factors recruit immune cells to the infection site, thereby amplifying the inflammatory response.

Z-FV7 not only combines effectively with LPS but also exhibits strong bactericidal properties. It can disrupt the bacterial membrane, allowing the cellular contents to be released, which leads to bacterial death. This process helps avoid the activation of inflammatory reactions in the body, both by Z-FV7 itself and by the LPS it produces [30].

Inflammatory factors play a vital role in activating the inflammatory pathway. Pro-inflammatory cytokines, such as *IL-1β*, *IL-6*, and *TNF-α*, are key mediators in activating the NF-κB pathway. These pro-inflammatory factors typically carry negative charges, while positively charged antimicrobial peptides can bind to them. This binding prevents the translocation of NF-κB, interrupts the phosphorylation processes of IκBα and P65, thereby inhibiting the activation of inflammatory pathways [10].

Additionally, other research has demonstrated that antimicrobial peptides can also bind to TLR4, which prevents LPS from binding and subsequently activating downstream pathways [31].

A significant aspect of the structural integrity of the endometrium relies on tight junction proteins, which consist of many kinds of proteins, including ZO-1 and claudin-1 [32]. LPS can damage these tight junctions and reduce the expression of these proteins, leading to increased transcellular and paracellular transport. This promotes the diffusion of cytokines and enhances the invasive effects of LPS on cells, ultimately worsening cell damage and inflammatory infections [33].

The results showed that Z-FV7 strengthens the tight junction structure of cells and normalises the expression of ZO-1 and claudin-1 proteins, helping to counteract the disruption of tight junctions by LPS. Tight junction-associated proteins are known to inhibit inflammation, and ZO-1 and claudin-1 appear to play similar roles in this process. However, the expression levels of ZO-1 were not significantly reduced in the antibiotic-treated group due to the effects of antibiotic treatment [34]. Unlike the untreated group, the expression levels in the antibiotic-treated group did not return to normal. This may be because antibiotics can negatively impact the protective role of normal flora and hinder the recovery of epithelial cells [35]. Beneficial flora have been shown to inhibit inflammatory responses and restore epithelial barrier function, highlighting the important role they play in maintaining cell integrity.

In the mouse model, Z-FV7 demonstrated significant bactericidal effects in vivo. It alleviated uterine congestion and oedema, reduced the bacterial load in the uterus, and decreased inflammatory cell infiltration, vacuolisation and collagen fibre rupture. These results were consistent with those obtained from in vitro assays. Additionally, the changes in the expression of cytokines and proteins related to the NF-κB signalling pathway mirrored those observed in the cell model. Overall, these findings indicate that Z-FV7 exhibits strong bactericidal activity both in vivo and in vitro.

In this experiment, we modified an antimicrobial peptide based on its antimicrobial principles, resulting in the design of the antimicrobial peptide Z-FV7, which features a ꞵ-hairpin structure and a positive charge. The antimicrobial peptide demonstrates effective bactericidal activity both in vivo and in vitro while exhibiting a favourable safety profile, failing to cause any harm to the organism. Additionally, it promotes the restoration of tight connection structures between cells and tissues. Notably, Z-FV7 shows promising therapeutic effects in treating endometritis caused by *E. coli*, outperforming traditional antibiotics. Compared with traditional antibiotics, the antibiotic also achieved better therapeutic effects in the treatment of endometritis caused by *E. coli*. Furthermore, the investigation into the anti-inflammatory mechanisms of this antibacterial peptide in the treatment of endometritis provides a theoretical foundation for its future clinical applications. It is hoped that this peptide can be widely adopted in the future to treat bacterial infections, potentially replacing traditional antibiotics.

## Data Availability

All the data generated or analysed during this study are included in this published article.
